# Polysomes of *Trypanosoma brucei*: Association with Initiation Factors and RNA-Binding Proteins

**DOI:** 10.1371/journal.pone.0135973

**Published:** 2015-08-19

**Authors:** Cornelia Klein, Monica Terrao, Diana Inchaustegui Gil, Christine Clayton

**Affiliations:** Zentrum für Molekulare Biologie der Universität Heidelberg, DKFZ-ZMBH Alliance, Im Neuenheimer Feld 282, D69120, Heidelberg, Germany; Universidad Nacional Autonoma de Mexico, MEXICO

## Abstract

We report here the results of experiments designed to identify RNA-binding proteins that might be associated with *Trypanosoma brucei* polysomes. After some preliminary mass spectrometry of polysomal fractions, we investigated the distributions of selected tagged proteins using sucrose gradients and immunofluorescence. As expected, the polysomal fractions contained nearly all annotated ribosomal proteins, the translation-associated protein folding complex, and many translation factors, but also many other abundant proteins. Results suggested that cap-binding proteins EIF4E3 and EIF4E4 were associated with both free and membrane-bound polysomes. The EIF4E binding partners EIF4G4 and EIF4G3 were present but the other EIF4E and EIF4G paralogues were not detected. The dominant EIF4E in the polysomal fraction is EIF4E4 and very few polysomal mRNAs are associated with EIF4G. Thirteen potential mRNA-binding proteins were detected in the polysomes, including the known polysome-associated protein RBP42. The locations of two of the other proteins were tested after epitope tagging: RBP29 was in the nucleus and ZC3H29 was in the cytoplasm. Quantitative analyses showed that specific association of an RNA-binding protein with the polysome fraction in sucrose gradients will not be detected if the protein is in more than 25-fold molar excess over its target binding sites.

## Introduction

Kinetoplastids are highly reliant on post-transcriptional mechanisms to control gene expression. There is strong evidence for transcriptome-wide control of mRNA translation [1,2] and decay [3], and also for variations in rates and efficiency of mRNA processing [3,4]. Numerous papers have documented the roles of RNA-binding proteins in all three processes [5–11]. The trypanosome genome encodes over 125 proteins with consensus RNA-binding domains. In a high-throughput RNAi screen, depletion of at least 60 of these was shown to result in a growth defect in at least one life-cycle stage [12].

We recently completed a genome-wide screen for proteins with potential activity as post-transcriptional regulators. The screen took advantage of the high-affinity interaction between the lambdaN peptide and its cognate recognition sequence, boxB. We identified protein fragments that could either enhance, or suppress, expression of the selectable markers when "tethered" via the lambdaN peptide to a reporter mRNA with boxB sequences in the 3'-untranslated region (3'-UTR). The screen identified about 300 potential regulatory proteins. These included thirty-nine proteins with RNA binding domains: 16 inhibited expression, and 23 enhanced it. Meanwhile, in a separate set of experiments, we found that a protein called MKT1 could enhance gene expression, probably via a complex that recruits poly(A) binding proteins [13]. MKT1 may associate with mRNAs via specific RNA-binding proteins: yeast 2-hybrid screens and affinity purification revealed several with potential to interact with MKT1 [13], some of which also showed activities in the tethering assay.

The results of our tethering screen also showed that some translation initiation factors can enhance expression—even when bound to the 3'-UTR. In general in eukaryotes, initiation factor eIF4E binds to the mRNA cap, and recruits eIF4G, which in turn recruits the helicase eIF4A and the 43S initiation complex. Trypanosomes have six variants of EIF4E and five of EIF4G [14–16]. Trypanosome EIF4E4 is known to associate with EIF4G3, while EIF4E3 can interact with EIF4G3 and EIF4G4 [14]. In *Leishmania*, EIF4E2 and EIF4E4 are partially associated with polysomes [17] but EIF4E4 is also strongly in the 80S fraction [18]. *L*. *major* EIF4E2 and EIF4E4 both also interact with the heavily methylated kinetoplastid cap structure. The role of EIF4E3 is somewhat enigmatic. *Leishmania* EIF4E3 migrated at 80S and bound the kinetoplastid cap poorly [17]. *T*. *brucei* EIF4E3 did not detectably bind to m^7^G, but binding to the modified trypanosome cap was not tested. RNAi targeting *EIF4E3* clearly inhibited both cell growth and protein synthesis, but it is not clear whether the effect on protein synthesis was direct or indirect [14]. *T*. *brucei* EIF4E6 interacts with EIF4G5 [16], while EIF4E5 interacts with EIF4G1 and EIF4G2 [15]; the roles of these factors in protein synthesis are currently unclear. In our *T*. *brucei* tethering screen, EIF4E3, EIF4E4, EIF4G2, EIF4G3, EIF4G4, and EIF4G5 were all identified as promoting reporter expression, whereas EIF4E1 suppressed expression (S2 Table in [19]).

Mammalian eIF4G can circularise bound mRNA through an interaction with poly(A) binding protein (PABP) [20], giving a closed loop that facilitates ribosome recycling, and there are similar reports from other organisms (reviewed in [21]). A recent study of 18 *in vivo* formaldehyde cross-linked yeast mRNAs, however, suggested that *in vivo* not all mRNAs are in closed loops mediated by eIF4G-PABP interactions [21]. There are also reports that eIF4G is not needed once translation has initiated [22,23]. It is not known whether kinetoplastid mRNAs can be circularised via such interactions. In *in vitro* pull-down assays, *L*. *major* EIF4G3 and 4G4 both interact with EIF4A1 [24], and the EIF4G3 interaction was seen using the yeast-2-hybrid assay [25]. *L*. *major* EIF4G3 interacts with PABP1 by pull-down [24] but not 2-hybrid [26], EIF4G4, in contrast, showed no such interaction. The 2-hybrid assay detected an interaction between PABP and EIF4E4 [26]. Trypanosome PABPs strongly enhance gene expression in the tethering assay [13,27].

We report here the results of experiments designed to identify RNA-binding proteins that might be associated with polysomes. After some preliminary mass spectrometry of polysomal fractions, we investigated the distributions of selected tagged proteins using sucrose gradients and immunofluorescence. Our results also yielded insights into potential roles of EIF4E3, EIF4E4, EIF4G3 and EIF4G4 in translation.

## Methods

### Trypanosomes and plasmids

Bloodstream-form and procyclic-form trypanosomes (Lister 427 strain) were grown as in HMI-9 and MEMPros media, with regular dilution such that densities varied from 5x10^4^ to 2x10^6^ cells/ml for bloodstream forms, and 5x10^5^ to 5x10^6^ cells/ml for procyclics. All relevant culture methods are described in detail in S1 Document. Trypanosome Genomic DNA was isolated using the illustra GenomicPrep cells and tissue DNA isolation kit (GE Healthcare). PCR reactions were done using GoTaq (Promega) polymerase or, for long open reading frames, Q5 Polymerase (New England Biolabs). Plasmids used for expression of tagged proteins, and oligonucleotides used for amplification are tabulated in S1 Table and S2 Table.

### Polyribosome isolation

Bloodstream-form trypanosomes (culture density 1x10^6^ cells/ml) were incubated for 5 min at room temperature with 20 μg/ml cycloheximide, then briefly chilled in a dry ice ethanol bath (without freezing) before harvesting by centrifugation. Procyclic forms (culture density 4x10^6^ cells/ml) were pelleted at room temperature, and resuspended in 1/20 of the former volume before addition of cycloheximide to 100 μg/ml. After 5min at room temperature, cells were chilled on ice and pelleted. The pellets were either used immediately, or frozen at -70°C in 1x Polyribosome buffer (10mM Tris (pH: 7.4), 10mM MgCl_2_, 300mM KCl) [28] containing 200mM Sucrose, 10% Glycerol and 100 μg/ml cycloheximide. Before preparation of cell lysates, frozen pellets were thawed on ice and washed twice with 1x Polyribosome buffer containing 200mM Sucrose.

To separate free and membrane-bound polysomes, the cell pellet was resuspended in detergent-free lysis buffer (1x polysome buffer with 200mM Sucrose, 1 tablet/5ml complete protease inhibitor (Roche, without EDTA), 2mM/ml DTT, 0.4mg/ml heparin, 10μg/ml leupeptin, and 100μg/ml Cycloheximide) and added to an equal volume of washed glass beads (300μg/ml, same buffer). The mixture was vortexed for 20min in bursts of 30sec separated by 10 sec on ice. Lysis was checked under a glass slide and free and membrane-bound fractions were prepared as described [29]. The pellet ("membrane-bound" fraction) was resuspended in lysis buffer containing 0,2% Triton-X-100, and resuspended by pushing through a syringe needle, and cleared by ultracentrifugation (4°C, 30,000g, 20min, Rotor: S45A). Detergent was also added to the supernatant ("free" fraction). For total polysomes, cells were lysed in NP40-containing buffer, and [28] and the lysate was cleared by centrifugation.

The cell extracts were loaded onto 15–50% or 15–55% sucrose gradients (in the same buffer without detergent), and centrifuged either at 150,000 g for 2,5h or at 164,000 g for 2h. The fractions were collected and the OD_260_ profile measured using an ISCO 160 gradient former. To concentrate the proteins, samples were diluted 1:4 with gradient buffer without sucrose and concentrated using Centricon tubes (Molecular cut-off: 100kDa). Polysomes remained intact during the concentration procedure, as judged by a repeat gradient centrifugation. If required, samples were precipitated with TCA. For mass spectrometry, we characterised preparations as follows: free polysomes from 5.6 x 10^9^ and 1 x 10^10^ bloodstream forms; membrane-bound polysomes from 1 x 10^10^ bloodstream forms; free and membrane-bound from 1.35 x 10^10^ procyclic forms. Results, as analysed using Scaffold S3, are in the S3 Table. Only proteins that were identified with greater than 97.5% probability were included.

For gel electrophoresis and Western blots, 3-4x.10^8^ cells were used for total polyribosomes and 2-3-fold more for the free and membrane-bound fractions. Fractions were concentrated before loading using microcon columns, as necessary. To calculate the percentages of each protein present in polysomes, the signals were quantitated, background was subtracted, then the remaining signal corrected according to the loading. The corrected signals for polysomes (at least two ribosomes) and the remainder (or for polysomes, monosomes and the remainder) were then added together, and the percentage of protein in each fraction, relative to the total, was calculated.

### Western blotting and immunofluorescence

For Western blotting of whole cell extracts, pellets of 0.2 - 1x10^7^ cells were used; for polysome gradients, portions of each fraction were used as indicated in Figures. Proteins were separated by SDS-PAGE, transferred to a nitrocellulose membrane, and stained with Ponçeau red. Antibodies used for protein detection were anti-V5 (Abgene), anti ribosomal protein S9 (raised against recombinant protein) and anti-trypanothione reductase (kind gift from Luise Krauth-Siegel, BZH, Heidelberg). Antibodies to EIF4E and G subunits were kindly given by Dr. O. de Melo Neto, Funcaçao Oswaldo Cruz, Brazil. Bands were labelled using the Western lightning Plus or Ultra ECL^T^ detection reagents (Perkin Elmer) and detected either by autoradiography or using the LSA-4000 imager.

Immunofluorescence was performed according as previously described [30]. Anti-myc and the anti-V5 antibodies from Abgene (mouse, 1:500) were used.

### RNA preparation and analysis

Total RNA was isolated from 2–10 **x**10^7^ cells at a densities of 0.5–1 x 10^6^ cells/ml for bloodstream forms and 1–6 x 10^6^ cells/ml for procyclics, using pegGOLD TriFast (Peqlab, GmbH). RNA was separated by formaldehyde gel electrophoresis, blotted onto Nytran N membranes, UV-cross-linked, and stained with methylene blue. Specific mRNAs were detected using ^32^P-labeled probes (Prime-It RmT Random Primer Labeling Kit, Stratagene) and detected by phosphorimaging.

## Results and Discussion

### The association of translation factors with polysomes

**Fig 1:**
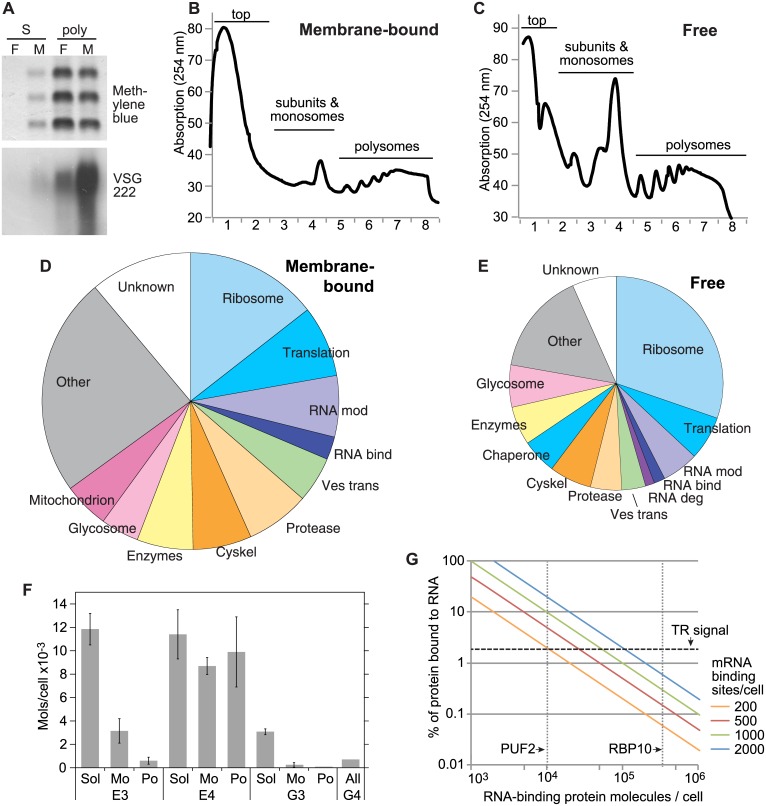
Association of EIF4E complexes with polysomes. A: Northern blot probed against the *VSG* mRNA with bloodstream-form samples made from the soluble protein peak (S) and the polyribosomes (poly) from the gradients shown in panel B (free, F) and panel C (membrane-bound, M), in order to show the proper separation of free and membrane-bound polyribosomes. B: Absorption at 254nm after sucrose gradient fractionation of a typical membrane-bound fraction from bloodstream-form trypanosomes. C: Absorption at 254nm after sucrose gradient fractionation of a typical free fraction from bloodstream-form trypanosomes. D,E Pie charts illustrating the protein functional classes in polysomal fractions in membrane-bound (D) and free polysomes (E). Results are pooled from bloodstream-form and procyclic-form parasites. All proteins were manually classified; see S3 Table for details. These diagrams show the results for 523 proteins, chosen using a threshold of >97.5% confidence and at least 2 peptides; to calculate the class enrichment, we used a unique gene list which includes only on representative of repeated genes (S3 Table sheet 2). The eleven most abundant categories are illustrated for each preparation; with the remainder grouped as "Other". Categories present in both preparations have the same colour code, and the areas of the circles are proportional to the total numbers of proteins identified. Ribosome: ribosomal proteins; Translation: translation factors and amino-acyl tRNA synthetases; RNA mod; RNA modification enzymes; RNA bind: proteins with RNA binding domains; RNA deg: components of the RNA degradation machinery; Ves trans: vesicular transport; Protease: proteases and peptidases; Cyskel: cytoskeletal proteins; Glycosome: glycosomal enzymes and components; Mitochondrion: mitochondrial proteins; Unknown: proteins of unknown function. F: Calculated numbers of molecules of EIF4E3, EIF4E4, EIF4G3 and EIF4G4 in the polysomes. Results are arithmetic mean and standard deviation from 3 (EIF4E3), 4 (EIF4E4), and 6 (EIF4G3) gradients. Sol = soluble fraction, Mo = subunits and monosomes; Po = polysomes. The total numbers of molecules per cell are taken as 1.5 x10^4^ (EIF4E3), 3 x10^4^ (EIF4E4) [14], 3400 (EIF4G3) and 700 (EIF4G4) [24,35]. In two experiments less than 5% of EIF4G4 was on polysomes but this is to small to show. G. The percentage of an RNA-binding protein that will be bound to RNA (“% bound protein”) was calculated relative to the protein abundance, for proteins that bind to 200, 500, 1000, or 2000 mRNAs per cell. On average, 2% of trypanothione reductase is found in the polyribosomes (data from six independent experiments).

Our initial aim was to identify the proteins associated with free and membrane-bound polysomes. Cells were suspended in isotonic buffer and lysed by agitation with glass beads before separation of free and membrane-bound fractions by centrifugation. Controls for bloodstream forms confirmed association of *VSG* mRNA with the membrane-bound fraction [29] (Fig 1A). After detergent addition, both fractions were layered onto sucrose gradients. During gradient harvesting we monitored the OD at 254nm in order to detect the polysomal fractions (Fig 1B and 1C). The signals indicated that for a given cell input we obtained approximately three times more free polyribosomes than membrane bound. Monosomes and ribosomal subunits were less abundant in the membrane-bound (Fig 1B) than in the free fraction (Fig 1C). The fractions were diluted and concentrated using Centricon columns with a 100kDa cut-off, both to reduce soluble protein contamination and to facilitate TCA precipitation.

Mass spectrometry of the polysomal fractions robustly detected 523 proteins (see Fig 1 legend and S3 Table). Fewer proteins were found in the free than in the membrane-bound fractions; 205 were present in both. Proteins connected with translation (ribosomal proteins, translation factors and tRNA synthetases) were prominent: 34% of all detected proteins in the free fraction, and 22% in the membrane-bound (Fig 1D and 1E). We detected all annotated ribosomal proteins except L1 and L39, several enzymes implicated in rRNA and tRNA modification including amino-acyl tRNA synthetases, both poly(A) binding proteins, all eight subunits of the TRiC nascent polypeptide folding complex, and RACK1 [31]. We also found four RNA helicases: they included DED1-1 and DED1-2, which are associated with translation [32], and the homologue of Brf1, a component of the yeast mRNP. Of the translation factors, EF1, EF2, EIF2, EIF5, the release factors, all subunits of EIF3 [33], and some subunits of EIF4 were found.

However, the preparations were also extensively contaminated with abundant cytosolic proteins, including components of the cytoskeleton, glycosome and (for procylic forms) the mitochondrion. Coomassie blue staining of gels from similar experiments indeed later suggested that the polysomal fractions contained roughly 10% of total cell protein (D. Inchaustegui, ZMBH, manuscript in prepration). Since many proteins associated with translation—including some translation factors and the ribosome—are very abundant, all of the results have to be regarded with caution in the absence of independent verification. Also, proteins in large structures are expected to be in the polysomal fraction. As expected, the membrane-bound fraction included many proteins involved in vesicular transport. Presumably these too are mainly contaminants, although some components of the translocon might be expected to remain polysome-associated after detergent treatment. We therefore decided not to repeat the mass spectrometry analysis but instead to examine the distributions of individual proteins. From now on, all experiments were done with bloodstream forms, and without any separation of free and membrane-bound fractions.

We first looked at the cap-associated initiation complex. EIF4A1 was clearly present, but of the other subunits, only EIF4E3, EIF4E4, and their binding partners EIF4G4 and EIF4G3, were detected. Moura et al. [24] suggested that in procyclic form trypanosomes, EIF4E3 and EIF4G4 are the major players in translation initiation. Analysis of polysomes from bloodstream-form *T*. *brucei*, however, revealed that only 4±2% of EIF4E3 was in the polysomes, which contrasted with 33%±10% of EIF4E4 (Fig 1F); using published abundances [14], this is a maximum of 4000 molecules per cell of EIF4E3 and 1.2 x 10^4^ molecules of EIF4E4 (Fig 1F). There are roughly 2 x 10^4^ mRNA molecules per bloodstream-form trypanosome, and 4.4 x 10^4^ per procyclic form [34]. Our preliminary results suggest that 70–80% of these mRNAs are in the polysome fraction (manuscript in preparation). It is therefore just about possible that in bloodstream forms, every polysomal mRNA is bound by an EIF4E—but this would mostly be EIF4E4.

In procyclic *T*. *brucei*, there are about 2 x 10^4^ molecules of EIF4G3 and 3000 EIF4G4 [24]. This is less than the number of mRNAs in procyclic-form polysomes. From quantitative mass spectrometry data [35] and the smaller cell volume, abundances in bloodstream forms will be even lower. Moreover, despite the clear polysomal association of EIF4E4, less 3% of its partner EIF4G3 was in the polysomal fraction of bloodstream forms (Fig 1F). This is consistent with two previous reports that eIF4G is not required for translation re-initiation on pre-existing polysomes in a HeLa cell *in vitro* translation system [22,23].

### Association of RNA binding proteins with polysomes

**Fig 2:**
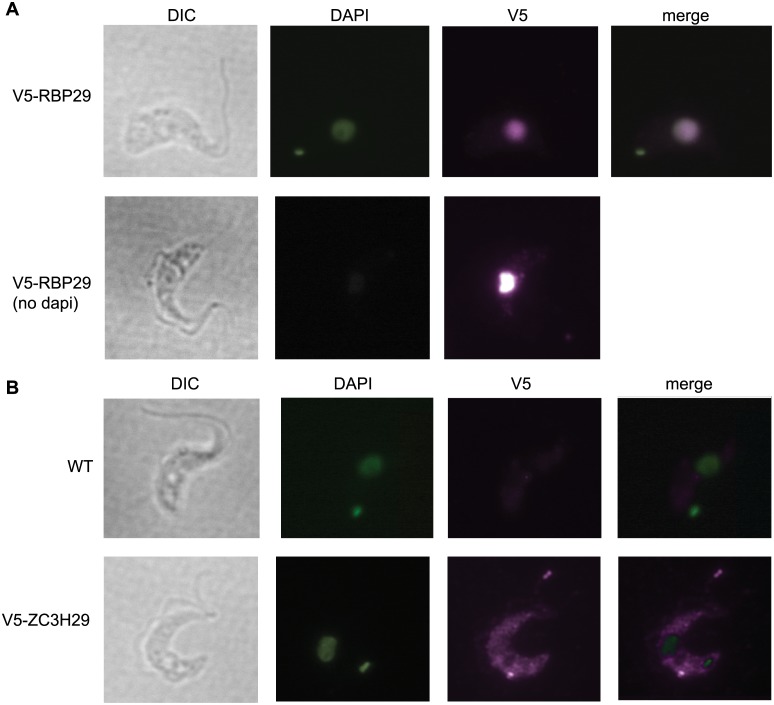
Locations of V5-RBP29 and V5-ZC3H29. (A) An *RBP29* gene was tagged *in situ* with a sequence encoding a V5 tag, in bloodstream forms. The tag was detected using anti-V5 antibody, with or without DNA staining with DAPI. Cells were shown as differential interference contrast (DIC) images. (B) Location of V5-ZC3H29 by immunofluorescence. Details as for (A).

Our principal aim was to find out which RNA-binding proteins could be detected in polysome fractions (Table 1 and S1 Table). Using a threshold of at least two peptides per protein, ten proteins with RNA-binding domains were unambiguously identified in only one preparation, RBP42 and ALBA4 were seen in two and DRBD3/PTB1 in three. (Table 1 includes three additional proteins for which only 1 peptide was detected.) To see if these proteins have an active capacity to influence mRNA abundance, we checked their activities in the published tethering assay [19]. Three of them—RBP42, DRBD3/PTB1, and RBP29—had been found to increase expression. More than 40% of all RBP42 is known to co-sediment with polysomes; and it binds to many different coding regions with no clear consensus recognition sequence [36]. DRBD3/PTB1 is mainly in the cytosol of unstressed cells [37], although some must be in the nucleus since it influences *trans* splicing [38]. DRBD3 stabilizes a subset of proteins in procyclic forms through binding to their 3'-UTRs [37]; it also binds to PABPs [39]. A preliminary analysis of procyclic-form polysomes, however, revealed that less than 2% of DRBD3 was in the polysomal fractions (not shown). In contrast, we found that V5-tagged RBP29 was mainly in the nucleus (Fig 2A) which is not consistent with a primary role in translation control.

**Table 1:** RNA binding proteins found in polysomal fractions. The numbers of peptides detected in each preparation are shown for all identified proteins with RNA-binding domains. The proteins are arranged in alphabetical and numerical order. mb = membrane-bound. Activity after tethering to reporter mRNAs [19] is denoted as ++ for a reliable increase in expression (satisfying stringent criteria as defined in [19]), + for a moderate increase,—for clear decrease and—for mild decrease; o = no effect.

Name	Gene ID	Domain	BS free	PC free	BS mb	PC mb	Tether Activity
ALBA4	Tb927.4.2030	ALBA	1	1	2	2	-
DRBD3/PTB1	Tb927.9.8740	RRM	2		5	2	++
RBP11	Tb927.8.4450	RRM	2				o
RPB29	Tb927.10.13720	RRM			3		++
RBP42	Tb927.6.4440	RRM	3		1	2	++
DRBD18	Tb927.11.14090	RRM	2				+
PUF1	Tb927.10.4430	Puf			2		-
PUF2	Tb927.10.12660	Puf			6		-
PUF3	Tb927.10.310	Puf			2		--
PUF6	Tb927.10.11760	Puf			4		o
PUF10	Tb927.11.6740	Puf	-	-	-	2	o
ZC3H18	Tb927.7.2140	CCCH			1		o
ZC3H29	Tb927.9.9520	CCCH			2		--
ZC3H30	Tb927.10.1540	CCCH			1		o
ZC3H32	Tb927.10.5250	CCCH			1		+
ZC3H41	Tb927.11.1980	CCCH				5	o
Whole fraction			1510	423	1860	2132	

BS: bloodstream form; PC: procyclic form.

Some of the RNA-binding proteins that were detected in one of the polysomal fractions suppressed expression when tethered to a reporter mRNA [19,40]. These include PUF1, PUF2. PUF3, and ZC3H29. V5-tagged ZC3H29 was in the cytoplasm (Fig 2B), which would be consistent with regulation of mRNA. We were, however, unable to confirm co-migration of *in situ-*V5-tagged ZC3H29 with polysomes. This was true whether or not the polysomes were separated into free and membrane bound fractions (S1 Fig, and data not shown). PUF1 co-migrates with monosomes [41], while PUF2 migrates at the top of a sucrose gradient {Jha, 2014 #2845). Tagged PUF3 showed no evidence of polysomal association (S2A Fig).

RNA-binding domain proteins with no clear activity in the tethering assay that were also detected in one preparation were DRBD18, ZC3H41, PUF6 and PUF10. DRBD18 (which we detected in bloodstream-form free polysomes) has both repressing and activating activities in procyclic forms, depending on the degree of arginine methylation {Lott, 2015 #3076}, Co-migration of PUF6 with polysomes could not be confirmed (S2B Fig). PUF10 is mainly in the nucleolus and is implicated in rRNA processing [42]; its presence in polysomal fractions could be due to residual association with ribosomes.

The *VSG* mRNA is expected to comprise at least 10% of the mRNA in membrane-bound polysomes of bloodstream forms. Six putative RNA-binding proteins were detected only in that fraction: PUFs 1, 2, 3, and 6, RBP29 and ZC3H29. To find out whether PUF3, PUF6, RBP29 or ZC3H29 binds to the *VSG* mRNA, tagged-protein-RNA-complexes were pulled down by immunoprecipitation, and *VSG* mRNA was assayed by RT-PCR. However, no clear association was seen (not shown).

### Polysome association can only be detected if the protein: target-RNA ratio is less than 25:1

The inconsistency between the proteomics and the Western blotting results for ZC3H29, PUF1, PUF3 and PUF6 prompted us to ponder the detection limits for association of a protein with polysomal mRNAs. As a baseline, about 2% of the negative control protein, trypanothione reductase, was in the polysomal fraction. Therefore, for a protein even to be considered as possibly polysomal, at least 4% of it would need to be found in the polysomes. We initially considered an RNA-binding protein X which, at steady state, is bound to 2000 different sites in polysomal mRNAs. For different abundances of protein X, we plotted the percentage of protein X that would be in bloodstream-form polysomes (Fig 1G, blue line). If there were 10^4^ molecules per cell of protein X, 20% of it would be in the polysomes: but if there were 10^5^ molecules of protein X per cell, the percentage in polysomes would only be 2%. So with 10^5^ molecules of protein X per cell, polysomal association would not be detected. We then repeated the calculation for three other numbers of binding sites (Fig 1G, green, red and yellow lines). Thus, if protein X bound to 500 sites, and there were 10^4^ molecules of protein X per cell, 5% of protein X would be polysome associated, but if there were 3x10^4^ molecules of protein X per cell, its polysomal association would not be detected. Fig 1G shows that Western blotting of gradient fractions, as described here, can demonstrate polysomal association of an RNA-binding protein only if the ratio of protein molecules to target mRNA binding sites is less than 25:1. In practice, however, the protein:RNA-target-site ratio needs to be lower because we cannot assume that every binding site will be occupied at all times.

We examined what this meant for two well-characterised proteins. RBP10 is relatively abundant [43] and could exceed the 2% threshold only it associated with every mRNA. PUF2 is present at about 10^4^ molecules/bloodstream-form trypanosome [40]. Given a single recognition site on each target mRNA, PUF2 would have to bind to at least 400 RNA molecules—or 2% of all mRNAs—in order for PUF2 association with polysomes to be double the background (Fig 1G). As a guide, there are 450 RNAs/cell that encode enzymes of glucose metabolism, 650 encoding chaperones, and a similar number for proteins involved in vesicular transport. In contrast, there are 250/cell for DNA metabolism and 100/cell for cell cycle regulation [3]. RBP42 is found on polysomes because it binds more than 1000 mRNAs in a relatively non-sequence-specific fashion [36].

Recently, much more specific methods have been developed to detect proteins that are associated with mRNAs. These procedures involve RNA-protein cross-linking, followed by mRNA purification with very stringent wash steps [44,45]. These methods are much more sensitive than gradient analysis and are likely to give a much more specific list of mRNA-bound proteins.

## Supporting Information

S1 Fig Most V5-ZC3H29 is not associated with polyribosomes. A: Polyribosome profile and Western blot using bloodstream cells expressing V5-ZC3H29. TR: trypanothione reductase, negative control S9: ribosomal protein S9 (small subunit), positive control. B: Summary of the quantified results for V5-ZC3H29 and TR, for three Western blots made with samples from three different gradients.
(PDF)

S2 Fig Puf3-TAP and Puf6-TAP are mostly not associated with polyribosomes. Trypanosomes expressing TAP-tagged PUF3 or PUF6 were lysed and polysomes were separated by sucrose gradient centrifugation. Protein was precipitated and analysed by Western blotting and detection of the TAP tag. A: Sucrose gradient profile for bloodstream-form cells expressing TAP-tagged PUF3. B: Quantification of Western blots, for the fractions shown in (A), after correction for different loading (not shown). TR: trypanothione reductase, negative control, S9: ribosomal protein S9 (small subunit), positive control. A second experiment gave a similar result. C: Sucrose gradient made with bloodstream cells expressing TAP-tagged PUF6. D: Summary of the quantified results of three Western blots made from samples of three different gradients, results shown as arithmetic mean ± standard deviation. The distributions of both PUF3 and PUF6 were similar if the extracts were treated with RNase (not shown).
(PDF)

S1 Table Plasmids. (DOCX)

S2 Table Oligonucleotides. (DOCX)

S3 Table Mass spectrometry results. The numbers of peptides found for each protein (at least 95% confidence) are shown. Proteins are included only if at least two different peptides were found in at least one preparation. Class designations were done manually. BS: bloodstream form; PC: procyclic form.
(XLS)

S1 Document Culture methods. (DOCX)
