# Supplementary figures and images for: Polysomes of Trypanosoma brucei: Association with Initiation Factors and RNA-Binding Proteins
Source: PLoS One. 2015 Aug 19;10(8):e0135973. doi: 10.1371/journal.pone.0135973 (PMC4545788; doi:10.1371/journal.pone.0135973)

**A**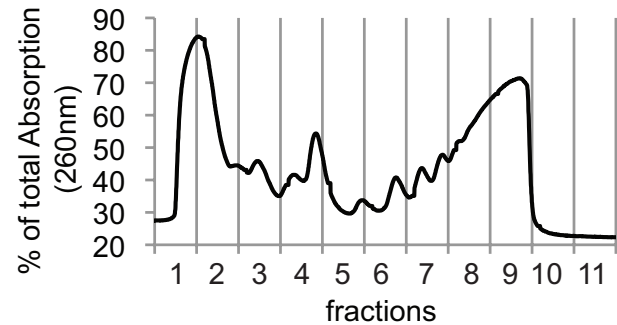

loading ( $\mu$ l)    3   10   10                      30  
fractions    1   2   3                      4   5   6   7   8   9   10   11

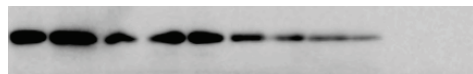

V5-ZC3H29

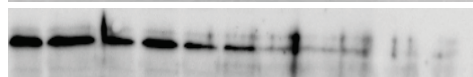

TR

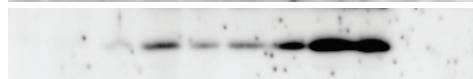

S9

**B**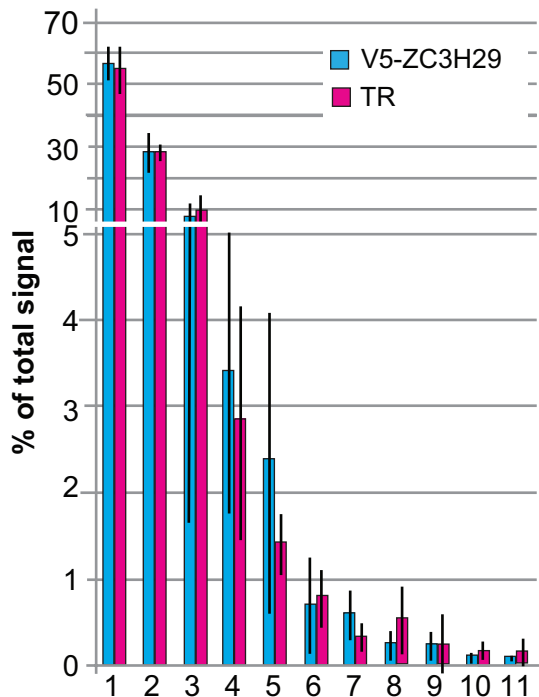

Supplement: S1 Fig — A: Polyribosome profile and Western blot using bloodstream cells expressing V5-ZC3H29. TR: trypanothione reductase, negative control S9: ribosomal protein S9 (small subunit), positive control. B: Summary of the quantified results for V5-ZC3H29 and TR, for three Western blots made with samples from three different gradients. (PDF) [file pone.0135973.s001.pdf]

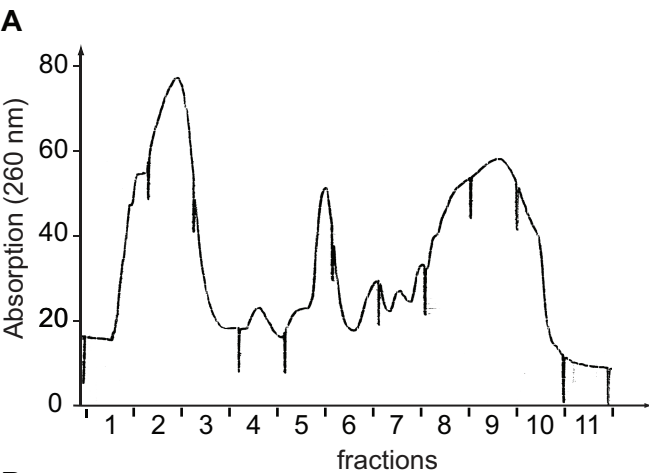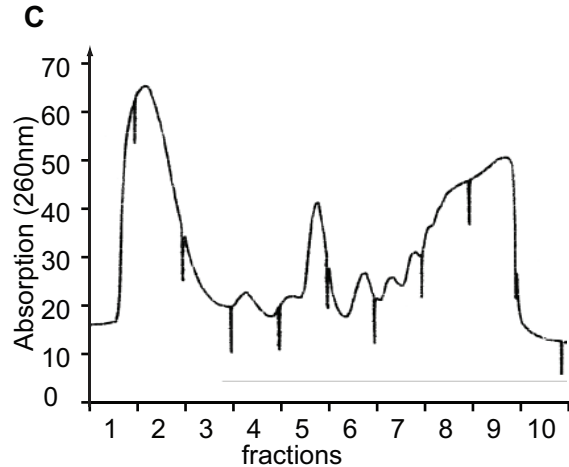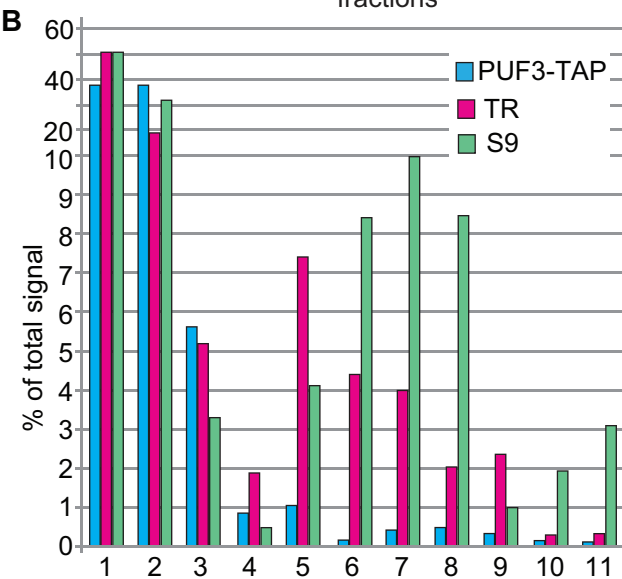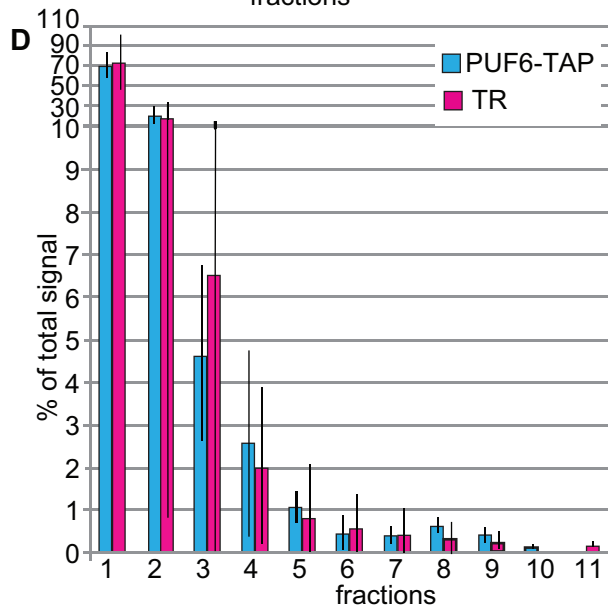

Supplement: S2 Fig — Trypanosomes expressing TAP-tagged PUF3 or PUF6 were lysed and polysomes were separated by sucrose gradient centrifugation. Protein was precipitated and analysed by Western blotting and detection of the TAP tag. A: Sucrose gradient profile for bloodstream-form cells expressing TAP-tagged PUF3. B: Quantification of Western blots, for the fractions shown in (A), after correction for different loading (not shown). TR: trypanothione reductase, negative control, S9: ribosomal protein S9 (small subunit), positive control. A second experiment gave a similar result. C: Sucrose gradient made with bloodstream cells expressing TAP-tagged PUF6. D: Summary of the quantified results of three Western blots made from samples of three different gradients, results shown as arithmetic mean ± standard deviation. The distributions of both PUF3 and PUF6 were similar if the extracts were treated with RNase (not shown). (PDF) [file pone.0135973.s002.pdf]
