# Supplementary material for: Polysomes of Trypanosoma brucei: Association with Initiation Factors and RNA-Binding Proteins
Source: PLoS One. 2015 Aug 19;10(8):e0135973. doi: 10.1371/journal.pone.0135973 (PMC4545788; doi:10.1371/journal.pone.0135973)
Supplement: S1 Table — (DOCX) [file pone.0135973.s003.docx]

**Supplementary Table S1**

**Plasmids**

| **pHD number** | **description** |
| --- | --- |
| 1781 | pHD1744 +PUF3 ORF (TAP-tag-HYG) |
| 1783 | pHD1744 +PUF6 ORF (TAP-tag-HYG) |
| 2161 | Bla V5 + RBP29 ORF + UTR |
| 2162 | Bla V5 + ZC3H29 ORF+ UTR |
| 2343 | Bla-V5 ZC3H32 ORF +UTR |
