# Supplementary material for: Polysomes of Trypanosoma brucei: Association with Initiation Factors and RNA-Binding Proteins
Source: PLoS One. 2015 Aug 19;10(8):e0135973. doi: 10.1371/journal.pone.0135973 (PMC4545788; doi:10.1371/journal.pone.0135973)
Supplement: S2 Table — (DOCX) [file pone.0135973.s004.docx]

### Supplementary Table 2

**Oligonucleotides**

| **CZ number** | **name** | **sequence** | **used for**  **pHD** |
| --- | --- | --- | --- |
| 3819 | rvORFZC3H29Apa1 | ATAgggcccGATAGCAACCGTGGAATA | 2162 |
| 3820 | fwORFZC3H29Xho1 | CAGctcgagATGACACAGGTCATCACG | 2162 |
| 3821 | fwUTRZC3H29Sac2 | CACccgcggATGTATTTGTTAAACGAG | 2162 |
| 3822 | rvUTRZC3H29Xba1 | CACtctagaGTTAATGGGCAATAACTA | 2162 |
| 3823 | fwORFrbp29Xho1 | GAGctcgagATGGCGCATGTTGGACAG | 2161 |
| 3824 | rvORFrbp29Apa1 | ATAgggcccTGCTCGCAGTATTGCTGG | 2161 |
| 3825 | fwUTRrbp29Sac2 | CACccgcggGAGCCTAGAAAATAGATA | 2161 |
| 3826 | rvUTRrbp29Xba1 | ATAtctagaCCGTTCGGCGCTTGCCTT | 2161 |
| 5438 | ZC3H28 fw Mlu1 | GCGacgcgttaTATTCTAGCGAAAAGGAGAAG | 2579 |
| 5437 | ZC3H28 rv Hpa1 | TATgttaacTCACGAACGACCCGGATGCTT | 2579 |
| 4678 | Z32UTRRvXbaI | GCGtctagaGATGTTTTCCTTCCCCTT | 2343 |
| 4679 | Z32UTRfwSac2 | CATccgcggACTCACTCATACATCTAA | 2343 |
| 4681 | Z32ORFrvApa1 | ATAgggcccAAGCCAGAATATCATCT | 2343 |
| 4682 | Z32ORFfwXho1 | CATctcgagATGTCTGGCACTAATCAC | 2343 |
