# Supplementary material for: Polysomes of Trypanosoma brucei: Association with Initiation Factors and RNA-Binding Proteins
Source: PLoS One. 2015 Aug 19;10(8):e0135973. doi: 10.1371/journal.pone.0135973 (PMC4545788; doi:10.1371/journal.pone.0135973)
Supplement: S1 Document — (DOCX) [file pone.0135973.s006.docx]

# 1. Cultivation of bloodstream trypanosomes

## 1.1. Large equipment

Gassed incubator (5% CO2) at 37°C, with humid atmosphere

Laminar flow hood

Liquid nitrogen tank for parasite storage

Inverted microscope

(preferably) normal microscope with phase-contrast optics

Filtration apparatus for sterilisation (may also be disposable)

Water bath for warming medium and heat-inactivation of serum

## 1.2. Small equipment and consumables

Neubauer counting chamber

Sterile flat-bottomed "T-flasks"

Rubber bulbs and pipette bulbs or electronic pipettors

500 ml sterile bottles for medium storage

Sterile pipettes in metal cans

Pasteur pipettes

Pipette tips, preferably with filters

Sealable plastic tubes for freezing

## 1.3. Medium

### Medium HMI-9

| Component | 100 x Stock | Amount | Final concentration |
| --- | --- | --- | --- |
| IMDM |  | 176.6g |  |
| sodium bicarbonate |  | 30.24 g |  |
| hypoxanthine | 13.6 mg/ml | 1.36 g | 1 mM |
| thymidine | 3.9 mg/ml | 390 mg | 0.16 mM |
| Bathocuproine sulfonic acid | 2.82 mg/ml | 282 mg | 0.05 mM |

Dissolve to a final volume of 10 l , filter sterilize and aliquot 450ml into bottles. Store at -20°C.

### Additional stocks for the medium

| Component | 100x Stock | Final concentration |  |
| --- | --- | --- | --- |
| Cysteine | 18.2 mg/ml | 1.5 mM | Filter sterilize, store no more than 1 month, -20°C |
| ß-mercaptoethanol | 14 µl /10ml |  | Filter-sterilize, make fresh |
| Heat-inactivated newborn calf serum (FCS) |  |  | heat to 56°C for 30 min, if consumption is not very rapid make 50 ml aliquots in sterile tubes and freeze |

Before use add to 450 ml medium:

5 ml each cysteine and ß-mercaptoethanol solutions

50 ml FCS

Note that powdered HMI-9 is available from laboratories working on trypanosomes in the UK.

For freezing: DMSO or glycerol.

References for trypanosome cultivation, including plating on soft agar, are (Carruthers and Cross, 1992; Duszenko et al., 1986; Hirumi and Hirumi, 1989). There is a detailed discussion of this topic at http://tryps.rockefeller.edu/DocumentsGlobal/tryp_culture_commentary.html

## 1.4. Culture method

Bloodstream trypanosomes of the Lister 427 strain can be cloned as single cells, with as few as 1 cell per ml. They grow to a maximum cell density of about 7 x 10^6^ / ml. It is very important not to exceed this as the cells will go into irreversible stationary phase and die. Ideally, do not exceed 2 x 10^6^/ml, then the cells will remain in log phase. If the density is higher before you dilute the culture, there may be a pause before growth resumes.

The cell density usually increases 10-20-fold in 24 hr.

The cells are aerobic and are grown in a 5%CO_2_ incubator at 37°C in flasks with loosened or vented caps.

Large volumes can be grown in sealed glass or plastic roller bottles in a warm room but in that case it is important that at least 80% of the volume should be air. This is easiest achieved by equilibrating the bottles for an hour in the incubator before tightening the caps. An alternative is to use multi-layered flasks.

## 1.5. Freezing bloodstream trypanosomes

To freeze the cells, take a growing culture (0.5 - 2 x 10^6^ cells/ml and add an equal volume of sterile medium containing 20% glycerol. Aliquot 0.5-1 ml of cells per tube, and cap tight. Make at least 3 tubes. Wrap the cells in paper tissues or wadding, with a thickness of about 5 cm. Place in the -70°C freezer. After 1-7 days place in liquid nitrogen. After a further day, remove a tube and thaw rapidly. Place into medium and incubate overnight to check viability and the ability to grow. Although parasite motility is one indicator of viability, it does not always guarantee that the parasite can grow. Do not throw away a growing culture until you are certain that the frozen stock is satisfactory. To recover cells it can help to try different densities and to omit selective drugs immediately after thawing. (Glycerol can inhibit growth.)

# 2. Cultivation of procyclic trypanosomes

## 2.1. Equipment

Incubator set to 27°C. Incubators normally have to be set to at least 5°C above the ambient temperature in order to maintain the temperature. Even in northern Europe, if your laboratories are not air-conditioned you will probably need a refrigerated incubator. Incubators or warm rooms used for *S. cerevisiae* can also be used so long as the temperature does not exceed 30°C.

(During the course we are going to improvise, by leaving the parasites on the bench!)

Laminar flow hood

Liquid nitrogen tank for parasite storage

Inverted microscope

(preferably) normal microscope with phase-contrast optics

Filtration apparatus for sterilisation (may also be disposable)

Water bath for warming medium and heat-inactivation of serum

## 2.2. Small equipment and consumables

Neubauer counting chamber

Sterile flat-bottomed "T-flasks"

Rubber bulbs and pipette bulbs or electronic pipettors

500 ml sterile bottles for medium storage

Sterile pipettes in metal cans

Pasteur pipettes

Pipette tips, preferably with filters

Sealable plastic tubes for freezing

For large volumes: conical flasks with stoppers (as for bacteria).

Filtration apparatus for sterilisation

## 2.3. Medium MEM-Pros:

| Component | g/10 litres | Component | g/10 litres |
| --- | --- | --- | --- |
| CaCl2.2H2O | 2.65 | L-Arg.HCl | 1.26 |
| KCl | 4.00 | L-Cys.Cys | 0.24 |
| MgSO4.7H2O | 2.00 | L-Gln | 2.92 |
| NaCl | 68.00 | L-His.HCl.H2O | 0.42 |
| NaH2PO4.H2O | 1.40 | L-Ile | 0.52 |
| HEPES | 71.40 | L-Leu | 0.52 |
|  |  | L-Lys | 0.73 |
|  |  | L-Met | 0.15 |
|  |  | L-Phe | 1.00 |
|  |  | L-Thr | 0.48 |
|  |  | L-Try | 0.10 |
| MEM non-essential amino acids | 100 ml | L-Tyr | 1.00 |
| MEM vitamins | 100 ml | L-Val | 0.46 |
| Phenol red (1000x) | 10 ml (or 0.1 g) | L-Pro | 6.00 |
|  |  | adenosine | 0.12 |
|  |  | ornithine.HCl | 0.10 |

Many labs order this or a similar medium as a ready-made powder.

Make the salt solution including HEPES in a volume of about 4 litres and adjust pH to 7.4 using NaOH. Add all the other solids and wait until dissolved; dilute up to about 9 litres as necessary. Add the MEM solutions, make up to final volume and sterile-filter. Leave out overnight to check for contamination. Store in aliquots of 450ml at 4°C.

### Additional stocks for the medium

| Component | Stock |  |
| --- | --- | --- |
| Hemin | 250mg of Hemin in 100ml 0.1M NaOH | Autoclave Aliquot into 10ml aliquots in sterile tubes and store at 4°C. |
| Heat-inactivated newborn calf serum (FCS) |  | heat to 56°C for 30 min, if consumption is not very rapid make 50 ml aliquots in sterile tubes and freeze |

Before use add to 450 ml medium:

50ml heat-inactivated foetal calf serum

1.5ml hemin solution.

For freezing: glycerol

## 2.4. Culture methods

Procyclic trypanosomes usually die if they are diluted below about 1 x 10^5^ cells /ml, although the presence of other cells can be mimicked by using conditioned medium. The component of conditioned medium that promotes parasite growth is not known. The parasites grow to a maximum cell density of 1-2 x 10^7^ / ml and they can be left for about a day at this density without dying, although such cultures may take a day or two to start growing after dilution. The cell density usually increases 4-fold in 24 hr although behaviour varies between lines and laboratories. The cells are aerobic and are classically grown at 27°C, but growth at 30°C is also possible. If the ambient temperature rises above about 22°C it will be necessary to buy a refrigerated incubator, as most incubators can only operate at temperatures 5°C above the ambient temperature. Note that the ability to grow at 30°C allows the parasites to share an incubator or warm room with *Saccharomyces cerevisiae*; if you do this contamination can be avoided simply by wrapping the caps of flasks with parafilm.

Small volumes are easiest to grow in plastic and large volumes can be grown in slowly-shaking glass conical flasks.

The parasites are aerobic, but the flasks cannot be left open in refrigerated incubators or warm rooms because the cultures will dry out. The solution to this is to make sure at least 80% of the volume is air and - for flat flasks- to lay the flask down to ensure a maximum air-liquid interface. Caps should then be screwed down tightly to avoid evaporation. When using 24- or 96-well plates, seal them with parafilm.

## 2.5. Freezing procyclic trypanosomes

Freezing and thawing protocols are exactly as for bloodstream forms but using the appropriate medium.

# 3. Transfection of bloodstream trypanosomes

## 3.1. Equipment

Electroporator capable of withstanding a pulse at 1.5kV with medium containing physiological salt

Bench-top centrifuge (up to 2000 rpm).

All other equipment and consumables for trypanosome culture

## 3.2. Consumables

5 ml flat culture flasks

24-well plates

50 ml and 15 ml sterile conical-bottomed tubes.

Electroporation cuvettes

## 3.3. Transfection medium (modified Cytomix)

| Component | Stock | How much | Final concentration |
| --- | --- | --- | --- |
| EGTA | 0.5M | 0.4 ml | 2 mM |
| KCl |  | 0.9 g | 120 mM |
| CaCl_2_ |  | 0.0017g | 0.15mM |
| ^a^KPO_4_ pH 7.6 | 0.1M | 10 ml | 10 mM |
| HEPES |  | 0.596 g | 25 mM |
| MgCl_2_.6H_2_O |  | 0.102 g | 5 mM |
| Glucose |  | 0.5 g | 0.5% |
| BSA (defatted) |  | 10 mg | 100µg/ml |
| ^b^hypoxanthine | 13.6 mg/ml | 1 ml | 1 mM |

^a^KPO_4_: Mix 8.66 ml 1M K_2_HPO_4_ with 1.34 ml KH_2_PO_4_ in 90 ml H_2_O.

^b^hypoxanthine: dissolve in 0.1M NaOH

Dissolve in 90 ml, adjust to pH 7.6 with 1M KOH, make up to 100 ml and filter-sterilize. Store at 4°C

## 3.4. Selective drug concentrations

| Drug | Stock | Amount for 10 ml | Final concentration |
| --- | --- | --- | --- |
| Blasticidin | 5 mg/ml | 10 µl | 5 µg/ml |
| G418 | 50 mg/ml | 1 µl | 5 µg/ml |
| Hygromycin | 50 mg/ml | 1 - 3 µl | *5 - 15 µg/ml |
| Phleomycin/Bleomycin | 5 mg/ml | 0.4 µl | 0.2 µg/ml |
| Puromycin | 1 mg/ml | 2 µl | 0.2 µg/ml |
| Tetracycline | 5 mg/ml in EtOH | 0.2 µl | 100 ng/ml |

*Ideal amount seems to depend on vector and cell line. Try with the lower amount first.

Stocks are stored in small aliquots at -20°C. Tetracycline should be kept frozen in the dark for no more than a month. The half-life of tetracycline in tissue culture medium is about 24 h so induction should be maintained by adding to 75 ng/ml every 2 days if no additional tetracycline-containing medium is added. An alternative is to initially add 1 µg/ml tetracycline; then the cells can be left for 4 days before re-adding the drug.

## 3.5. Preparation (Day 1)

1. Cut vector (10 µg/cuvette) with *Not*I. Check 0.2 µg of the digest on a gel. Heat (65°C, 10 min) to denature the *Not*I then ethanol precipitate.

2. Wash the precipitate with 70% ethanol, then with 100% ethanol. Fill the tube with the ethanol to sterilize it. In the tissue culture hood, remove the 100% ethanol with a sterile pipetted, spin again and (in the hood) remove the visible liquid. Then leave the tube(s) open in the laminar flow hood to dry.

3. In the hood, resuspend the DNA in 25 µl (per 10 µg) of sterile water or Tris-EDTA buffer. Make sure the DNA is well dissolved, heating if necessary. Store at -20 °C.

4. The day before transfection, inoculate fresh medium with trypanosomes at a final density of 10^5^/ml. You will need 1-2 x 10^7^ trypanosomes per transfection, and it is best to do two transfections for each DNA. Thus for each transfection you need 10-20 ml of culture at 10^6^/ml. Be sure also to allow for no-DNA and empty (*Not*-linearized) plasmid controls!

## 3.6. Transfection (Day 2)

1. On the day of transfection, check cell numbers and wait if necessary until minimum numbers are attained.

2. Centrifuge the trypanosomes at about 2000 rpm in a standard cell culture centrifuge and resuspend in ice-cold cytomix (1 ml per transfection, keep in the refrigerator). Set out the electroporation cuvettes on ice, thaw the DNA, and label recipient flasks. If the electroporation cuvettes come with a small Pasteur pipette, you should leave it, sterile, in the wrapper. Array the lids open-side-down on the sterile work surface in the laminar flow hood.

3. Re-centrifuge the cells, and resuspend in 0.5 ml cytomix per cuvette. Keep on ice. During the centrifugation, aliquot 5 ml of growth medium per flask. We also use 36 ml of medium (in a 25 cm flask – kept upright).

4. Aliquot the trypanosomes into the cuvettes, on ice.

5. For each transfection: Take two cuvettes from the ice, and place them on the work surface in the hood. Dry them carefully with tissue. Add 10 µg of cut DNA. Mix up and down with a Pasteur pipette or using a Gilson pipetman. It is critical that the DNA should be well mixed. Put on the cap.

6. Electroporate both cuvettes using settings of 1.5 kV and resistance R2. The time constant should be about 0.3 and there will be a little foaming. If there is a flash and bang, use the cells anyway, the transfection may still have worked.

7. Transfer the contents of each cuvette to a separate flask with medium.

8. Proceed, 2 cuvettes at a time, until all transfections are complete.

9. Take two flasks at random and count the cell density. It should be no more than 5 x 10^5^/ml and there should clearly be dead cells and debris. Dilute if necessary.

10. Place in the incubator overnight.

## 3.7. Selection and cloning (day 3)

1. Count the cells in each flask.

2. Dilute to 2 x 10^5^/ml and add selective drug. In fact, in most cases there’s no risk of overgrowth and the cells don’t need counting before adding drug and putting in multi-well plates.

3. Plate the cells out into two 24-well plates with a 1 ml per well. Any remaining culture can be kept in a larger flask. The no-DNA control can also be left in a flask.

## 3.8. Identifying transformants

1. Incubate the plates or flasks for 5 days. At this time, any well containing live trypanosomes should be recognizable under the inverted microscope. Upon seeing the parasites, count to determine the cell density. It is also easy to see when the parasites start to grow because the medium turns yellower. The controls are usually dead after 4 days, often earlier.

2. When the density reaches 10^6^/ml, transfer to a larger volume.

3. If there is no visible growth after 8 days throw the cultures away and start again.

4. If this happens three times, and a positive control experiment with empty vector gives clones, the transfected plasmid is probably deleterious to cell growth. For example, RNAi could be lethal even at low expression levels (such as leakage of the inducible T7 promoters). Under these circumstances you may wish to use an alternative region of the gene, or switch to the stem-loop procedure if not already doing so.

# 4. Transfection of procyclic trypanosomes

## 4.1. Equipment

Electroporator capable of withstanding a pulse at 1.5kV with medium containing physiological salt

Bench-top centrifuge (up to 2000 rpm).

All other equipment and consumables for trypanosome culture

## 4.2. Consumables

5 ml flat culture flasks

24-well plates or 96-well plates

50 ml and 15 ml sterile conical-bottomed tubes.

Electroporation cuvettes

## 4.3. Transfection medium ZPFM:

|  | Amount | Final concentration |
| --- | --- | --- |
| NaCl | 7.714 g | 132 mM |
| KCl | 0.596 g | 8 mM |
| Na2HPO4 (anhyd) | 1.136 g | 8 mM |
| KH2PO4 (anhyd) | 0.204 g | 1.5 mM |
| MgOAC.4H2O | 0.107 g | 1.5 mM |
| Ca0Ac2  OR CaCl_2_ | 0.016 g | 90 µM |
|  | 0.014 g |  |

Dissolve in 800 ml, adjust pH to 7.00+/- .05 with NaOH or HAc and make up to 1 l. It is also possible to make a 5x stock for storage at 4°C.

## 4.4. Selective drug concentrations

| Drug | Stock | Amount for 10 ml | Final concentration |
| --- | --- | --- | --- |
| Blasticidin | 5 mg/ml | 20 µl | 10 µg .ml |
| G418 | 50 mg/ml | 3 µl | 15 µg/ml |
| Hygromycin | 50 mg/ml | 10 µl | 50 µg/ml |
| Phleomycin/Bleomycin | 5 mg/ml | 1 µl | 0.5 µg/ml |
| Puromycin | 1 mg/ml | 10 µl | 1 µg/ml |
| Tetracycline | 5 mg/ml in EtOH | 0.2 µl | 100 ng/ml |

For details see section 2.3.1.2

## 4.5. Preparation (Day 1)

1. Cut vector (10 µg/cuvette) with *Not*I. Check 0.2 µg of the digest on a gel. Heat (65°C, 10 min) to denature the *Not*I then ethanol precipitate.

2. Wash the precipitate with 70% ethanol, then with 100% ethanol. Fill the tube with the ethanol to sterilize it. In the tissue culture hood, remove the 100% ethanol with a sterile pipetted, spin again and (in the hood) remove the visible liquid. Then leave the tube(s) open in the laminar flow hood to dry.

3. In the hood, resuspend the DNA in 25 µl (per 10 µg) of sterile water or Tris-EDTA buffer. Make sure the DNA is well dissolved, heating if necessary. Store at -20 °C.

4. The day before transfection, inoculate fresh medium with trypanosomes at a final density of 5 x 10^5^/ml. You will need 1-2 x 10^7^ trypanosomes per transfection, and it is best to do two transfections for each DNA. Thus for each transfection you need 10-20 ml of culture at 10^6^/ml (or smaller volumes if the culture is denser). Be sure also to allow for no-DNA and empty (*Not*-linearized) plasmid controls!

## 4.6. Transfection (Day 2)

1. On the day of transfection, check cell numbers and wait if necessary until minimum numbers are attained. You can transfect at densities up to, but not exceeding, 5 x 10^6^/ml.

2. Centrifuge the trypanosomes at about 2000 rpm in a standard cell culture centrifuge. After centrifuging the trypanosomes, transfer the supernatant (used medium) to a new sterile tube or bottle. Store it in the refrigerator. If you are worried about sterility you can re-filter the supernatant.

3. During centrifugation, set out the electroporation cuvettes on ice, thaw the DNA, and label recipient flasks. If the electroporation cuvettes come with a small Pasteur pipette, you should leave it, sterile, in the wrapper. Array the lids open-side-down on the sterile work surface in the laminar flow hood.

4. Resuspend cells in ice-cold ZPFM (1 ml per transfection, keep in the refrigerator).

5. Re-centrifuge the cells, and resuspend in 0.5 ml ZPFM per cuvette. Keep on ice. During the centrifugation, aliquot 5 ml of growth medium per flask. We also use 36 ml of medium (in a 25 cm flask – kept upright).

6. Aliquot the trypanosomes into the cuvettes, on ice.

7. For each transfection: Take two cuvettes from the ice, and place them on the work surface in the hood. Dry them carefully with tissue. Add 10 µg of cut DNA. Mix up and down with a Pasteur pipette or using a Gilson pipetman. It is critical that the DNA should be well mixed. Put on the cap.

8. Electroporate both cuvettes using settings of 1.5 kV and resistance R2. The time constant should be about 0.3 and there will be a little foaming. If there is a flash and bang, use the cells anyway, the transfection may still have worked.

9. Transfer the contents of each cuvette to a separate flask with medium.

10. Proceed, 2 cuvettes at a time, until all transfections are complete. Do not ever plan to do more than 10 different DNAs (20 cuvettes) in one set of electroporations.

11. Take two flasks at random and count the cell density. It should be between 2 and 8 x 10^5^/ml and there should clearly be dead cells and debris. Dilute if necessary.

12. Place in the incubator overnight.

## 4.7. Selection and cloning (Day 3)

1. Count the cells in each flask

2. Dilute the cells to 2 x 10^5^/ml using the conditioned medium that you saved from yesterday. Add selective drug.

3. Plate on a 24 well plate. You can either put 1ml of the culture per well, or make four serial two-fold dilutions (using the conditioned medium),. If you are targeting the vector into the rRNA locus, the transfection efficiency is often sufficiently low that plating the original culture is sufficient to give clonal populations. If you are targeting into a pol II locus the efficiency may be substantially higher and limiting dilution will be necessary.

## 4.8. Identifying transformants

1. Procyclic transfections usually need to be incubated for 7 days. At this time, any well containing live trypanosomes should be recognizable under the inverted microscope. Upon seeing the parasites, count to determine the cell density. It is imperative to count the cells as the appearance of the deep cultures in wells is deceptive and excessive dilution at this stage will kill the parasites. If there are no cells growing well after 10 days throw away the plates and start again.

2. When the density reaches 2 x 10^6^/ml, transfer to a larger volume but do not under any circumstances dilute to less than 5 x 10^5^/ml. Culture very carefully and as soon as you have a 5 ml culture, freeze a sample.

3. If the majority of wells is positive you may not have clones. This can if necessary be corrected later by re-cloning using conditioned medium or using normal (non-drug-resistant) cells as "feeders".

**5. Useful references**

Brun, R. and Schönenberger, M. (1979) Cultivation and *in vitro* cloning of procyclic culture forms of *Trypanosoma brucei* in a semi-defined medium. 36, 289-292.

Carruthers, V.B. and Cross, G.A.M. (1992) High efficiency clonal growth of bloodstream- and insect-form *Trypanosoma brucei* on agarose plates. *Proc. Natl. Acad. Sci. USA*, 89, 8818-8821.

Duszenko, M., Ferguson, M.A.J., Lamont, G., Rifkin, M.R. and Cross, G.A.M. (1986) Cysteine eliminates the feeder cell requirement for cultivation of *Trypanosoma brucei* bloodstream forms in vitro. *J. Exp. Med.*, 162, 1256-1263.

Hirumi, H. and Hirumi, K. (1989) Continuous cultivation of *Trypanosoma brucei* bloodstream forms in a medium containing a low concentration of serum protein without feeder cell layers. *J. Parasitol.*, 75, 985-989.

van Duersen, F.J., Shahi, S.H., C.M.R., T., Hartmann, C., Guerra-Giraldez, C., Matthews, K.R. and Clayton, C.E. (2001) Characterisation of the growth and differentiation *in vivo* and *in vitro* of bloodstream-form *Trypanosoma brucei* strain TREU 927. *Mol. Biochem. Parasitol.*, 112, 163-172.

Ziegelbauer, K., Quinten, M., Schwarz, H., Pearson, T.W. and Overath, P. (1990)

Synchronous differentiation of *Trypanosoma brucei* bloodstream to procyclic forms in vitro. *Eur. J. Biochem.*, 192, 373-378.
